# High-throughput microcontact printing of proteins in microwell cell culture plates

**DOI:** 10.1016/j.mex.2024.102665

**Published:** 2024-03-12

**Authors:** Daryan Chitsaz, Timothy E. Kennedy

**Affiliations:** Department of Neurology and Neurosurgery, Montreal Neurological Institute, McGill University, Canada

**Keywords:** High-throughput microwell microcontact printing, Micropatterning, Cell assay, High-content

## Abstract

Microcontact printing (MCP) is used to pattern a surface with a specific compound, allowing the spatially restricted response of cells to be assayed as they encounter a molecule of interest. MCP is a relatively low-cost and accessible technique that uses commercially available reagents and common cell culture equipment. However, it can be technically challenging, slow, and incompatible with microwell cell culture plates that are widely used for screening and other applications. Here, we describe a novel protocol using medical biopsy punches to transfer patterns into standard 96-well plates via polydimethylsiloxane (PDMS) cutouts. We demonstrate that this method can be used to deposit patterns of poly-D-lysine (PDL) into the microwells of glass-bottom plates. As a proof-of-concept, we show that cultured rodent glial cells preferentially grow and extend processes on the pattern. This method will allow larger scale MCP experiments in which different patterns, proteins, or other factors can be assayed in parallel.

• Biopsy punches enable both cutting out small circular stamps and plunging them into tissue culture microwells to transfer proteins.
• Compared to standard MCP, this method offers a more rapid workflow to pattern proteins onto substrates, and allows use of microwell plates that permits larger-scale experiments.

## Graphical abstract

**Figure:**
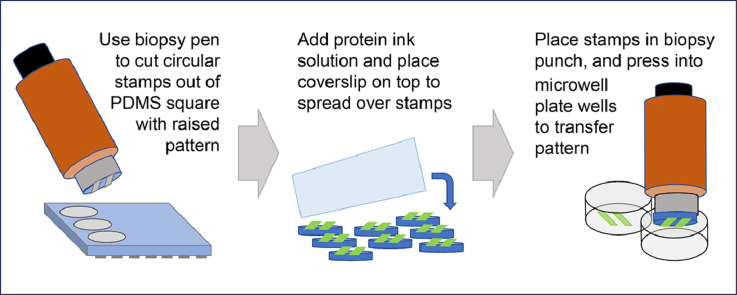

Specifications Table

**Table:** 

Subject Area:	Neuroscience
More specific subject area:	Biomedical Engineering
Name of your method:	High-throughput microwell microcontact printing
Name and reference of original method:	Sébastien G. Ricoult, Greta Thompson-Steckel, James P. Correia, Timothy E. Kennedy, David Juncker. Tuning cell–surface affinity to direct cell specific responses to patterned proteins. Biomaterials, Volume 35, Issue 2, 2014, Pages 727–736. https://doi.org/10.1016/j.biomaterials.2013.10.023
Resource availability:	NA

**Method details**

## Introduction

MCP is a form of soft lithography in which compounds such as proteins are deposited onto a surface in a specific pattern, generally using a polydimethylsiloxane (PDMS) stamp. This provides a powerful in vitro tool to evaluate how environmental cues, such as proteins presented by the extracellular matrix, regulate cell biology. Many proteins and peptides have been microcontact printed, including extracellular matrix molecules like fibronectin and collagen and guidance cues like ephrins and netrin-1 [1], [2], [3]**.** Substrate patterning has been used to study a variety of cellular mechanisms including adhesion, migration, and differentiation [4], [5], [6]. By curing PDMS on rigid master moulds, often created through silicone photolithography, stamps can be generated with micro- or nano-scale features such as stripes or dots [7]. Concentrated protein solutions can then be adsorbed onto these stamps, after which they are partially dried with non-reactive nitrogen gas and then pressed onto a cell culture substrate. MCP is typically performed on high-energy glass surfaces which can be “activated” with plasma gas treatment, though methods also exist to pattern polymers like polystyrene [8]. After transferring the pattern of protein, the substrate can be immersed in a solution with coating reagents such poly-D-lysine (PDL) where it adsorbs onto the exposed glass around the printed protein, creating a backfilled “reference surface” to support cell adherence [5]. This allows investigators to compare cells cultured on a standard reference surface substrate with the printed protein, and study how cells react as they extend or migrate to encounter the printed protein of interest, such as to determine whether it inhibits or promotes growth or process extension [5].

**Fig. 1:**
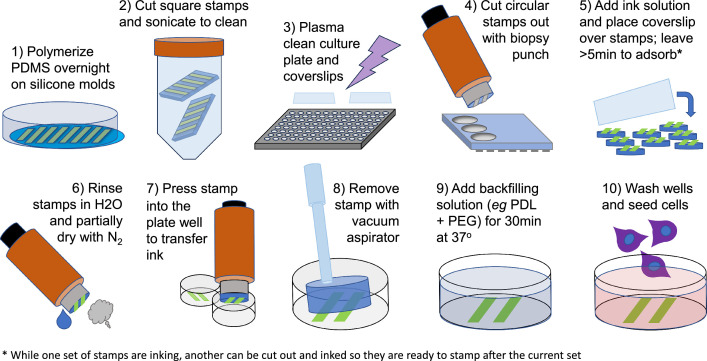
Schematic of the microwell MCP workflow.

**Fig. 2:**
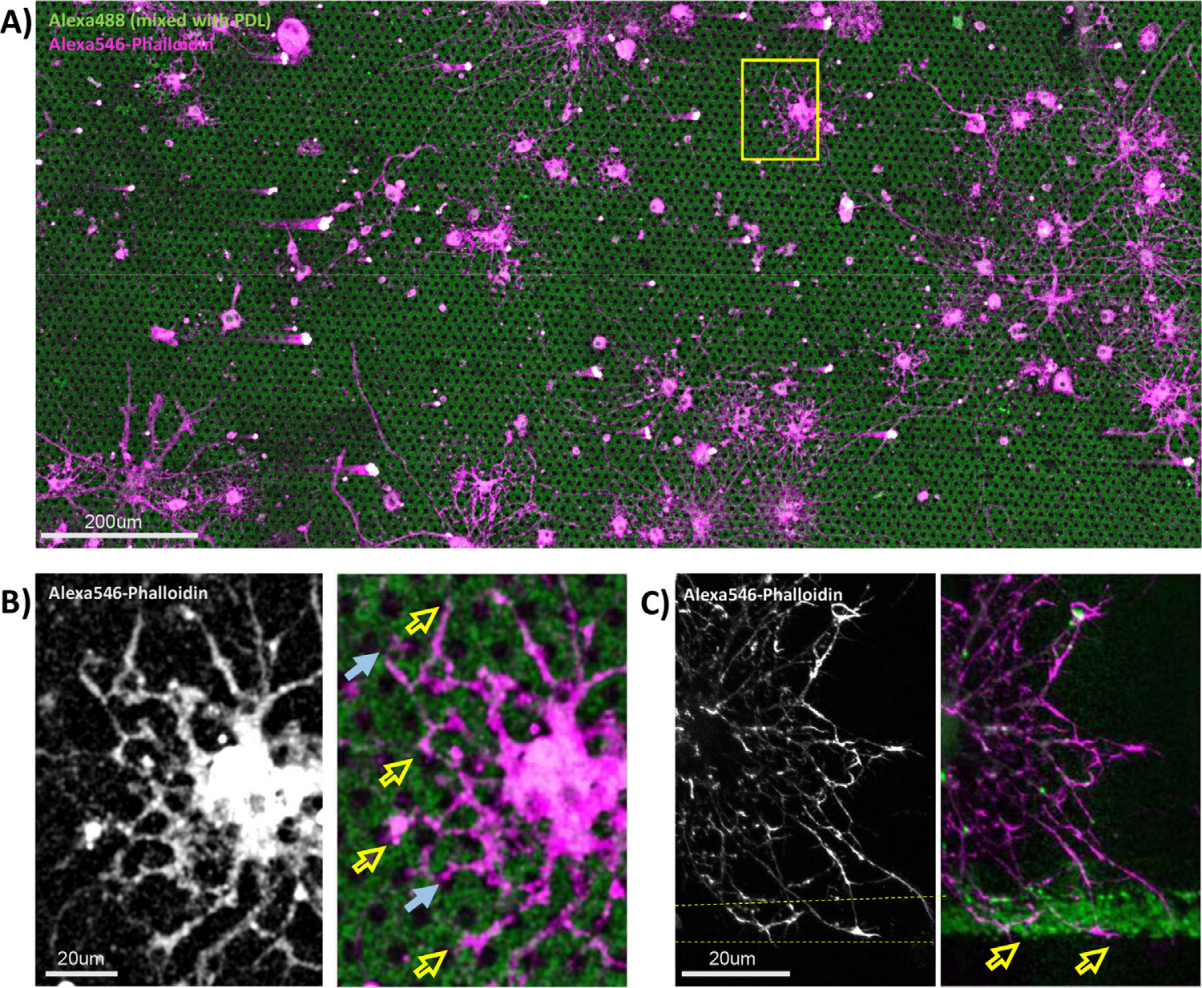
A) Representative well in a 96-well plate stamped with 10 µg/mL PDL mixed with 0.5 µg/mL Alexa488-conjugated IgG (green) in a honeycomb pattern with 5 µm circular holes, with a 50% PDL-50% PEG backfilled reference surface. Neonatal rat glia were cultured for 7 days and labeled with Alexa546 conjugated Phalloidin to visualize F-actin along cellular processes (magenta). B) Magnified image of a glial cell corresponding to the box in A. Some cellular processes can be seen growing over the holes (blue arrows), while most grow around on the more adhesive PDL pattern (yellow arrows). C) A glial cell from a different plate printed with 10 µm-thick stripe of PDL with a 50% PDL-50% PEG reference surface. Instead of growing outward, once encountering the stripe, processes deflect to extend along the edge of the comparatively more adhesive PDL stripe substrate (yellow arrows).

Our modified method for MCP utilizes biopsy punches, pen-like devices with extendible “hole punch” circular blades that can cut out stamps ideally sized for small tissue culture plate wells. The punches include extendible plungers that can press the stamp into the wells once they are inked. Stamps need to ink for at least 5 min, so it is possible to stagger sets of stamps by inking one set while the former is being printed such that printing can be done continuously. With this method, investigators can ink sets of 8 or more stamps at a time and pattern entire 96-well plates in approximately 1 h. A schematic with the major steps is shown in Fig. 1. An example image of a printed pattern of PDL with a fluorescent marker are displayed in Fig. 2.

## Protocol summary

1) **PDMS stamp fabrication.** Sylgard 184 is cast over silicone wafer moulds with etched pattern; after curing the PDMS is removed, cut into square stamps, and sterilized.
2) **Print preparation**. A biopsy punch is used to cut out a set of small circular stamps, and concentrated “ink” of a protein-of-interest mixed with a fluorophore, such as a fluorescent secondary antibody, is placed on top of the stamps in a humidified chamber and allowed to adsorb onto the PDMS surface.
3) **Culture device patterning.** The cell culture plate/device is treated in a plasma-cleaner, and the biopsy punch used to lift stamps and press them into the well to print several wells at a time, after which the stamps are aspirated out and a coating solution can be added to backfill the portion of the cell culture substrate that did not receive the stamped pattern.
4) **Cell culturing and imaging.** Cells are seeded into the wells, grown, fixed, and immunolabeled. Analysis of cell growth and expression of different markers on or off patterns can then be performed on resulting microscope images (Fig. 2).

## Equipment

- 10 cm diameter silicone wafer master moulds with etched patterns of interest, which can be used >20 times to make PDMS stamps
  ○ Moulds can be made in-house via photolithography, or ordered from a vendor (University Wafer, Boston, MA); alternatively low-cost moulds can be fabricated without needing a clean room, as described by Khadpekar et al. [9].
  ○ Features should be arranged in squares that are ∼1.5–2 cm^2^, allowing at least eight 0.5 cm diameter circles to be cut out
- 250 mL cylindrical beaker and spatula to mix PDMS
- Vacuum chamber
- Plasma cleaner (if unavailable see Karimian et al. [10])
- Bath sonicator (Branson 1510 Ultrasonic Cleaner)
- Sterile culture hood with vacuum aspirator
- Sterile forceps to manipulate stamps
- Nitrogen tank with blow gun to partially dry stamps
- Multiwell pipettor and aspirator, to add and remove solutions from 8 wells at a time (recommended)

## Reagents

- Sylgard 184 Elastomer Kit (Dow Chemicals)
- 15 cm diameter polystyrene dishes (ThermoFisher)
- Scotch tape
- Sterile milliQ water
- Sterile phosphate-buffered saline (PBS)
- 70% ethanol
- 50 mL falcon tubes (ThermoFisher)
- Sterile 10 cm petri dishes (ThermoFisher)
- Glass-bottomed cell culture plate to print onto, i.e. 96-well glass-bottom plate (Cellvis #P96-1.5H-N)
- Sterile biopsy punch (5 mm diameter to fit in 96-well plate well; World Precision Instruments #504528)
- 22 mm^2^ coverglasses (ThermoFisher #12-553-466)
- Concentrated protein solution to print (50 ug/mL in PBS or other buffer), or polymer like poly-D-lysine (Sigma #P4957) or dendritic polyglycerol amine, dPGA (DendroTEK Biosciences; [11])
- Alexa488-conjugated IgG antibody to mix with printed protein to label it (alternatively can immunolabel printed protein after fixation; ThermoFisher #A-11001)
  ○ Adding a fluorescent label as a fiduciary marking is not essential, but facilitates monitoring the fidelity of the printed pattern. For example, if the stamp is pressed down obliquely, more protein may be transferred to one side of the well
- Sterile glass or plastic Pasteur pipettes (to aspirate stamps out of wells; ThermoFisher #13-678-20C)
- 4% paraformaldehyde fixative solution (dissolved in PBS; Sigma #158127)
- Triton X-100 detergent (Sigma #X100-100ML) to permeabilize cells for intracellular labelling
- Fluorescence staining reagents (i.e. Alexa647-conjugated Phalloidin; ThermoFisher #A12379)
1) **PDMS stamp fabrication**
  1.1) Tape silicone wafer master onto the bottom of a 15 cm tissue culture dish with Scotch tape with features facing up, taping around the edges such that no solution can get under the silicone wafer when the polymer solution is added.
    ○ Keep the 15 cm dish closed when not using to prevent dust from landing; clean off with nitrogen gun before using.
  1.2) Vigorously mix Sylgard 184 encapsulant with the curing agent at a 10:1 ratio for several minutes until it takes on a homogeneous frothy appearance and pour over dish
    ○ Use a designated beaker and spatula; these will become coated with polymerized PDMS and will be unusable for other applications.
    ○ The volume of PDMS solution is very important – the resulting PDMS gel after curing should be thin enough to be cut by the biopsy punch, but thick enough to be able to reach the bottom of the microwell when it is being pressed in by the biopsy punch plunger. We found 22 mL in a 15 cm dish to be ideal, but the volume may differ depending on the plate well height and biopsy punch model.
  1.3) Leave dish in vacuum chamber overnight to draw out bubbles and have the elastomer cure.
  1.4) The next day, cut out square stamps small enough to fit in a 50 mL falcon tube for cleaning (∼1.5–2 cm^2^).
  1.5) Put square stamps in a 50 mL falcon tube filled with 70% ethanol and sonicate for 5 min to sterilize and clean off any excess oils.
    ○ Stamps can be stored in 70% EtOH at room temperature until ready for use.
2. **Print preparation**
  ○ All steps should be carried out in a sterile BSC.
  ○ This section describes how to print sets of 8 wells at a time, enough for 1 column of a 96 well plate.
  2.1) Place stamps in an open 15 cm dish in BSC to dry while preparing other reagents. Be sure all EtOH is evaporated from the stamps before they are used.
  2.2) Clean kimwipes by spraying with EtOH and leave in BSC to dry; these will then be wet with dH_2_O and placed in dishes along with stamps later to keep them humid and prevent drying.
  2.3) Fill two 50 mL falcon tubes with sterile PBS and dH_2_O and put in easily-accessible rack in BSC, to use later to dip the stamps in to rinse off excess ink solution.
  2.4) Open nitrogen tank valve and wipe down gun nozzle with 70% EtOH before placing in the BSC, where it will be used to partially dry the stamps.
  2.5) Put square coverglasses (1 for each set of 8 stamps) in petri dish, open in plasma chamber, and plasma treat for 1 min.
    ○ Keep track of which surface of each coverslip was face up in the cleaner; this side should be in contact with the ink.
  2.6) Plasma clean glass-bottomed 96-well plate for 1 min.
  2.7) Put a square stamp in a petri dish with the pattern facing up. Using the biopsy punch cut out 8 circular stamps and place them close together in another dish with the pattern still facing up.
    ○ May be helpful to use an illuminated magnifying glass, which can be taped to the glass of the BSC, to visualize which side of the stamp has the pattern.
  2.8) Prepare 10 µL of ink solutions for each circular stamp by mixing protein with 2 µg/mL fluorophore-conjugated antibody and diluting in a buffer like PBS.
    ○ E.g. to print 24 wells of a protein, 3 1.5 cm^2^ stamps and 260 µL of solution should be prepared (leaving 20 µL for pipetting error).
    ○ For 260 µL of 50 µg/mL PDL ink, we dilute 13 µL of 1 mg/mL PDL with 0.26 µL of 2 mg/mL IgG-488 in PBS.
  2.9) Ink first set of stamps by putting 10 µL of solution on top of each one, and then place a coverglass overtop to evenly distribute the solution over the pattern. Leave for 5 mins.
    ○ Add a folded sterile kimwipe with 2–3 mL of water added on top to the corner of the petri dish and keep lid closed – this provides a humidified chamber in which the ink can adsorb onto the PDMS without drying out.
    ○ Protect the dish from direct light by putting something opaque on the lid, such as covering with sterile aluminum foil.
  2.10) While the first batch of stamps is inking, the next set can be prepared; it takes ∼3–4 mins to deposit 8 stamps. By the time the last set has been printed the next set will be ready.
3. **Culture device patterning**
  3.1) For each stamp, place it back into the biopsy punch with forceps with the inked pattern facing out.
    ○ Can also invert the coverglass with all the stamps still on it – the surface tension of the ink should hold them in place if the coverglass on top of them is lifted and rotated, such that the patterns are now facing down. They can then be easily picked up by the biopsy punch.
  3.2) Dip the biopsy punch tip loaded with the stamp in PBS and then dH_2_O for ∼3 s each, and then dry off with a low pressure blast of nitrogen for ∼3 s.
    ○ Goal is to only remove any visible beads of liquid, not to totally dry stamp out; this may require some trial and error to establish the right timing.
  3.3) Place biopsy punch in plate well and press down lightly with plunger until you feel the stamp contacting the plate base.
    ○ Hold down with that amount of pressure for 5 s and then lift the punch back out and eject the stamp into a waste container.
    ○ The stamp will often remain attached in well and can be left there temporarily.
  3.4) Repeat for the remaining stamps.
  3.5) Use pipette and aspirator to remove any stamps left in wells, and add the backfill reference surface coating solution (e.g. PDL/PEG in PBS; [5])– can now ink the next batch, and start stamping one that was previously inked.
    ○ Patterned proteins should not be left to dry; if no backfilled reference surface coating is to be applied the patterned substrate should still be left in PBS.
    ○ The bioactivity of some patterned proteins may be better preserved in other buffers such as HBSS; this can be used for coating and washes instead of PBS.
  3.6) After the last well has been printed, incubate the plate at 37°C for at least 30 mins for the PDL (or other reference surface coating) to adsorb to the substrate.
  3.7) Wash 3 × 5 min with 100 µL of PBS per well with multiwell pipette and aspirator.
4. **Cell culturing and imaging**
  4.1) Prepare suspension of cells in desired culture media. For example, here we used neonatal rat oligodendrocytes in SATO media with a modified shake-off isolation procedure as previously described [12,13].
  4.2) Cell seeding density depends on the goal of the experiment, but it is advisable to use a relatively low density so that cells are well spaced out so their interactions with the protein pattern aren't confounded by contact with other cells.
    • For post-mitotic cells like glia we seed 10 000 cells/well at most for 96 well plates; for studies with actively dividing cells, like the HEK293 cell line, it is advisable to seed fewer (we found that seeding 2 500 HEK293 cells/well of a 96 well plate and culturing for 1 day yielded a good density).
  4.3) Culture cells in 200 µL of media/well; patterns typically remain stable for at least 2 weeks, but this will depend on the specific characteristics of the protein or polymer that is printed.
  4.4) Fix cells by removing culture medium, optionally washing with PBS to remove residual medium, and adding 100 µL of 4% PFA at 37 °C per well for 10 min.
    • Glutaraldehyde and other fixatives may be used to better preserve filamentous actin (F-actin) for phalloidin staining, as described [14].
    • In lieu of fixation, live cells can be imaged with brightfield microscopy or fluorescent dyes or proteins, visualizing how the cells interact with the printed proteins.
  4.5) Wash out fixative 3 × 5 min with PBS.
    • The plate can then be stored at 4°C wrapped in parafilm to prevent evaporation; with PBS at this temperature, surfaces patterned with many commonly used proteins and polymers are typically stable for at least 6 months.
  4.6) Optionally permeabilize cells with 0.05% Triton-100X in PBS for 10 min and then label cells with antibodies, dyes or other labeled molecular probes (e.g. Alexa647 linked phalloidin to mark F-actin).
    • Do not label using a fluorophore that is the same colour as that used for MCP. Additionally, because the pattern may be bright enough to bleed into other channels it may be helpful to use a distinct wavelength like Alexa647, or dilute the fluorophore in the ink to reduce brightness.
  4.7) Image with an inverted fluorescence microscope which allows the substrate in a cell culture plate to be visualized.
  4.8) Analyze cell growth or process extension on verses off the pattern, and at patterned edges, such as visualizing how local cellular morphology is affected and whether the subcellular distribution or post-translational modification status of immunolabeled proteins of interest may be locally altered.

## Conclusion

The development of a relatively high-throughput microcontact printing method to pattern tissue culture microwells offers a promising avenue to scale up typical MCP experiments for applications ranging from assaying drugs that alter cellular responses to printed cues to screening different substrates and patterns for biointerfaces [15]. Microwell plates offer a number of advantages over other cell culture substrates, like coverslips, including compatibility with high-content screening microscopes and allowing investigators to use fewer cells and reagents per well. Other methods to perform high-throughput printing are not suitable for microwell plates and require advanced microfabrication techniques that are inaccessible for most biomedical research labs [16,17]. Our method employs standard cell biology equipment and reagents and can be adapted for labs without access to plasma cleaners [10] or silicone wafer master moulds [9], making it highly accessible.

## Ethics statements

For primary glial cultures taken from neonatal rats, all procedures were performed in accordance with the Canadian Council on Animal Care guidelines for the use of animals in research.

## CRediT authorship contribution statement

**Daryan Chitsaz**: conceptualization, methodology, investigation, experiments, writing - original draft. **Timothy E. Kennedy**: funding acquisition, supervision, methodology, writing - review & editing.

## Declaration of competing interest

The authors declare that they have no known competing financial interests or personal relationships that could have appeared to influence the work reported in this paper.

## Data Availability

Data will be made available on request.
